# Metabolic Effects of Androgen-associated Body Mass in Klinefelter Syndrome

**DOI:** 10.21767/1989-5216.1000257

**Published:** 2018-02-18

**Authors:** MD Ehrhart, IR Guthrie, F Qeadan, MR Burge

**Affiliations:** Department of Internal Medicine & Endocrinology, University of New Mexico Health Sciences Center, Albuquerque, NM, USA

**Keywords:** Klinefelter syndrome, Testosterone, Androgen deficiency, Androgen replacement, Body mass index, Glucose intolerance

## Abstract

Patients with Klinefelter Syndrome (KS) are at increased risk for both diabetes and cardiovascular disease. While the anabolic effects of androgen replacement therapy may be associated with weight gain in such patients, the metabolic effects of this weight gain are unknown. Since untreated KS represents a natural example of androgen deprivation, we hypothesized that KS patients who are receiving androgen replacement would have a healthier metabolic risk factor profile, in addition to an increased Body Mass Index (BMI), relative to patients who are not receiving androgen replacement.

Using de-identified data collected from Health Facts (a national, consolidated, and relational database of Electronic Health Records), we identified 2,447 adult patients with an ICD-9 billing code for KS. Of these, 262 patients were included in this study based on available anthropometrics, metabolic profiles, and information about androgen replacement. Multiple linear regression analysis was performed using BMI as the dependent variable in a model that included age, androgen replacement therapy (yes or no), A1C, blood pressure, and fasting lipids. Post-hoc comparisons were made using frequency analysis and the unpaired Student’s t-test.

There were 81 patients with KS who received androgen replacement and 181 patients who did not. In multiple regression, only androgen therapy was positively and significantly associated with BMI while adjusting for other risk factors (p=0.03). Post-hoc comparison of metabolic risk factors revealed no other differences between patients who received androgen replacement and those who did not.

These data suggest that androgen replacement therapy in Klinefelter Syndrome is associated with increased BMI, but this increase does not appear to exert a detrimental effect on other metabolic risk factors in this condition.

## Introduction

Klinefelter syndrome (KS) is a congenital chromosomal abnormality causing primary hypogonadism attributable to the presence of one or more extra X chromosomes in male patients [1]. Prevalence estimates for KS vary between 450 to 700 cases per 100,000 individuals [1,2]. The most common karyotype is XXY, but cases with more than two X chromosomes, as well as complex mosaicisms, are also known to exist [3].

A prototypical synopsis of the syndrome comprises a tall, eunuchoid male who has narrow shoulders and broad hips, sparse body hair, gynecomastia, small testicles, androgen deficiency, and azoospermia [4]. Androgen deficiency in KS patients is often treated with androgen replacement therapy, but the wider metabolic implications of this therapy are not known.

In a previous study of androgen deprivation that involved patients with both KS and advanced prostate cancer, type 2 diabetes mellitus (T2D), and the metabolic syndrome were found to be more common than expected [4]. Additionally, sex hormone binding globulin (SHBG) concentrations were found to be independently associated with poor glycemic control in men with T2D, while total serum testosterone was not [4].

Other studies, however, have conflicted with these results, suggesting that testosterone levels and glycemic control were related [5,6]. Since SHBG and testosterone concentrations are both affected by exogenous testosterone (a common treatment for KS), it is important to determine how androgen replacement therapy might affect the overall metabolic status of KS patients [7].

It is possible that testosterone replacement therapy confers beneficial effects in KS patients suffering concurrently from other common metabolic disorders, such as T2D. In hypogonadal men with T2D, testosterone replacement correlates with improved glycemic control and reduced triglyceride levels [8].

Conversely, acute testosterone deficiency has been shown to produce a rapid increase in adinopectin production (a proposed mediator of insulin sensitivity in adipocytes), while testosterone supplementation in androgen-deficient males correlates with decreased adiponectin production [9]. Taken together, these findings suggest that although androgen therapy may have beneficial effects in individuals already suffering from metabolic imbalances, the mechanism through which it accomplishes these effects is poorly understood.

Testosterone supplementation in eugonadal males has been found to be associated with dose-dependent increases in fatfree mass, as well as decreases in fat mass and HDL cholesterol [10]. While this study was performed in healthy males, it suggests that androgen therapy may have mixed effects on the risk of obesity in addition to metabolic syndrome. [10]. Further, this change in body composition would decrease central obesity and may thus decrease rates of insulin resistance in this population. Since KS patients are known to be at increased risk for these conditions, the metabolic effects of androgen replacement therapy in KS patients deserve careful characterization [11].

In summary, while patients with KS are known to be at increased risk for many endocrine diseases, including the metabolic syndrome, T2D, autoimmune disease, and metabolic bone disease, the response of these diseases to androgen therapy in the specific setting of KS has been little studied.

We here describe a cross-sectional retrospective study to explore how testosterone replacement therapy affects glucose homeostasis status, lipid profile, blood pressure, and BMI in KS patients receiving androgen replacement therapy as compared to those who are not receiving androgen replacement therapy. We hypothesize that KS patients who are receiving androgen replacement therapy will have a healthier metabolic risk profile (despite an increased BMI) than those who are not taking androgen replacement.

## Methods

### Study subjects

Study patients were selected from the Cerner Health Facts database using a standard database query from the years 2000–2015. Health Facts is a national, consolidated, relational database of de-identified Electronic Health Records from 698 hospitals that employ the Cerner System (Cerner Corporation, Kansas City, MO, USA), and contains records from more than 60,000,000 unique individuals.

Using an ICD-9 billing code for KS during outpatient encounters, we identified 2,447 possible adult patients for inclusion in this study. This study was approved as exempt by the UNM HSC Human Research Review Committee.

### Study protocol

Study inclusion criteria comprised males between the ages of 18 and 60 years, inclusive, with an ICD-9 billing code for KS. Patients were excluded if they did not have a full medication list, since this made it impossible to determine whether or not they were receiving androgen replacement therapy.

Data collected for each patient included age, sex, race, ethnicity, BMI, height, weight, medication list, systolic blood pressure (SBP), diastolic blood pressure (DBP), A1C, fasting blood glucose, fasting lipid panel, thyrotropin (TSH), and free T4 concentrations. In all cases, the most recent available clinical data were collected for the purposes of this study.

### Cohort definitions

The Androgen cohort includes patients who had received androgen replacement over the prior 12 months relative to the date of the most recent ICD-9 billing date for KS. Androgen therapy was defined as an extant prescription for testosterone on the patient’s medication list for the purposes androgen replacement. The No Androgen cohort includes patients who had not received androgen replacement over the prior 12 months.

### Statistical analysis

To explore the associations between metabolic indices and androgen supplementation, multiple linear regression analysis was performed using BMI as the dependent variable in a model that included age, androgen replacement therapy (yes or no), A1C, blood pressure, and lipids as independent variables.

Direct statistical comparisons were made between the two groups using the unpaired Student’s t-test for continuous variables, which are reported as mean ± standard deviation [12,13]. Categorical variables were compared using the Chi-Square or Fisher Exact tests, which are reported as frequencies or relative frequencies [14]. Analysis was performed using SAS Version 9.4 (Cary, NC).

## Results

There were 262 patients identified with sufficiently complete data for inclusion in the study analysis. Specifically, 2,447 patients with KS were initially identified by ICD-9 code, 1,232 were eliminated because they were outside the inclusion age range of 18 to 60, and 953 were eliminated because they did not have a valid medication list, leaving 262 subjects for inclusion in the analysis.

There were 81 patients in the Androgen cohort and 181 patients in the No Androgen cohort. As shown in Table 1, the groups were similar with respect to age, race and ethnicity. Table 1 also shows that the Androgen cohort had a significantly higher mean BMI than did the No Androgen cohort (p=0.007). However, when separated into BMI category, as shown in Table 2, this statistical difference was diminished.

Summary results of metabolic indicators for both groups are shown in Table 3. There were no statistically significant differences observed in these metabolic parameters between patients who received androgen replacement therapy and those who did not. Specifically, no statistically significant differences were observed with respect to age, A1C, total cholesterol, LDL or HDL cholesterol, triglycerides, SBP, TSH, Free T4, or BMI category. Differences in DBP approached statistical significance (p=0.08), with patients who were not receiving androgen replacement therapy having slightly higher mean values (89 *vs.* 85 mmHg).

Additionally, there were no differences between the cohorts with respect to glucose homeostasis status (i.e. normal glucose homeostasis, prediabetes, or diabetes as defined by A1C), as shown in Table 4.

In the multiple regression analysis, it was confirmed that androgen replacement therapy was the only statistically significant independent predictor of BMI in this study (p=0.03) while adjusting for all other variables included in the study.

## Discussion

In this retrospective cohort study, we explored how androgen supplementation affected metabolic indicators in KS. Despite the acknowledged limitations of our data set, the results of this study suggest that androgen replacement therapy in KS was associated with an increase in BMI without changes in other metabolic indices.

From the multiple regression model, this was found to be a true independent association even after adjusting for possible confounders, including age, race, glycemic status, lipids, and blood pressure. A previous study found that testosterone caused an increase in fat free mass with a decrease in body fat mass, suggesting that the observed BMI differences in our study were possibly caused by a similar change in body composition [10]. Moreover, increases in bone mass that have been associated with testosterone supplementation might further explain this gain in body mass [15]. Unfortunately, we do not have access to body composition data or bone mineral density determinations in this study, so we are unable to comment upon specific mechanisms for the gain in body mass that we observed. Furthermore, given our somewhat limited data set, we cannot comment upon other conditions that may or may not be associated with androgen therapy and increased body mass in Klinefelter Syndrome.

A study by Bojesen et al. compared KS patients receiving testosterone replacement therapy to healthy controls, and they included a post-hoc analysis of treated versus untreated KS patients [11]. At odds with the current study, they found no differences between the groups with respect to anthropometric measures. Their primary finding was that both treated and untreated KS patients are at a higher risk for the metabolic syndrome than healthy controls, suggesting that testosterone therapy may positively impact abdominal adiposity and LDL cholesterol concentrations in KS. The current study confirms a high prevalence of metabolic syndrome in KS patients, as both cohorts met criteria for metabolic syndrome, on average, with glucose intolerance and hypertension. Conversely, although our data set is limited, we found no evidence that the prevalence of prediabetes or diabetes were affected by testosterone treatment nor that LDL cholesterol or triglyceride concentrations were increased by testosterone replacement.

To date, no randomized control trials and relatively few other studies have addressed the metabolic effects of testosterone replacement therapy in adult KS patients [11,16,17]. One systematic review of hypogonadal men with type 2 diabetes showed that testosterone replacement therapy was associated with benefit in glucose homeostasis and triglyceride levels while noting that larger RCTs are needed [8]. However, despite the biologic plausibility, the few extant KS-specific, non-RCT studies have generally shown that testosterone replacement therapy is associated with clear changes in body mass composition changes without strong accompanying benefit in insulin sensitivity or lipid profile [11,16,17]. The current study adds support to recent studies regarding the unhealthy metabolic profiles often observed in KS patients, as well as the lack of benefit to androgen therapy regarding the non-BMI components of the metabolic profile [6,8,11].

The strengths of our study include additions to the knowledge base regarding the treatment effects of a relatively rare disease, the use of the large Cerner Health Facts database, and the fact that our multiple regression analysis accounted for some of the common covariates and factors that may complicate any evaluation involving BMI. Additionally, our use of the Cerner Health Facts database allowed us to study a rare disease that cannot easily be studied using data from a single institution. Moreover, the multicenter, national scope of this database minimizes geographic bias based on race or other factors, making our results more generalizable to the entire United States KS population. An additional strength of our study is that any inadvertent “misclassification” of Klinefelter Syndrome patients as controls in our analysis would only induce a bias that tends to deflate our observed associations with BMI, and not the other way around. Hence, we would expect the true association with BMI to be even stronger in the KS population. Similarly, we have accounted for possible selection bias by adjusting our results by age and race.

Our study also had some weaknesses, including the fact that our cohorts were relatively small. Although there were 2,447 patients in the Health Facts database that possessed an ICD-9 billing code for KS, many of these patients did not have a medication list as part of their patient information. This information was essential to include patients in the current study since we used the medication list to assign patients to the correct group. This limited our study to only 262 patients; approximately 11% of potential available patients. Additionally, many of these patients did not have all of the laboratory test covariates that were included in the multiple regression analysis. As an example, only 73 out of 262 total patients had A1C values obtained over the previous 12 months in the dataset, and we were thus only able to assign glycemic status on a subset of the patients. Therefore, given the low number of patients included in A1C analysis, the conclusion of no difference in glycemic status between groups may not be a strong one. Further, the current study was not able to address differences in bone mineral density between groups, as noted by a previous study [18]. Health Facts does not currently include results of DXA Bone Mineral Density determinations in a manner that allows query, so we were unable to compare rates of osteoporosis or osteopenia between the groups. Additionally, our dataset does not include karyotype information, so we were unable to account for the possibility of KS mosaicism. According to a recent comprehensive review of Klinefelter Syndrome, however, karyotype analysis is required for a diagnosis of the condition [19]. Although we can reasonably assume that the patients described in this study received a karyotype analysis at some point in their lives, it is probable that many of these diagnoses were made during childhood, and since our study included only adults, it is not feasible to locate all of these results with the current study design.

Subgroup analysis could have offered additional conclusions about the metabolic phenotype of KS patients that may vary depending upon the penetrance of the genetic disorder [20]. Additionally, it should be noted that we do not have any information about the possible inclusion of more complex sex chromosomal pleiotropy in these patients (i.e. 47XXYY, 48XXXY, 49XXXXY, etc.) [21]. As such, all patients with an ICD-9 billing code for KS were analyzed as a single homogeneous diagnosis in the current study. Finally, since this was a cross sectional study, we defined testosterone replacement therapy as a prescription for testosterone residing on the medication list within one year of the KS ICD-9 designation. This method does not account for the dose or duration of therapy prior to laboratory testing. To ensure the validity of our laboratory assessments, however, laboratory assessments were included in the analysis only if they were obtained after the initiation of testosterone therapy for patients in the androgen replacement group.

Future research should expand the current study to include assessment of skeletal integrity, especially with respect to osteoporosis and osteopenia, should these data become available. Finally, the current study might be expanded to include investigation into how rates of autoimmune diseases are affected by testosterone replacement therapy [22].

In conclusion, in this cohort of adult patients with Klinefelter Syndrome from throughout the United States, the increase in BMI associated with testosterone replacement therapy did not adversely affect the metabolic profile.

## Figures and Tables

**Table 1:** Descriptive characterisitics of study participants.

Variable	Androgen Receiving (n=81)	No Androgen (n=181)
Age (years) (n=262)	41 ± 14	40 ± 13
BMI (kg/m^2^) (n=227)	34.7 ± 9.3	31.4 ± 7.6*
Race/Ethnicity (n=262)		
White, Hispanic and Non-Hispanic	67 (82.7%)	147 (81.2%)
Black, African American	6 (7.4%)	23 (12.7%)
Other (Asian, Pacific Islander, Native American)	1 (1.2%)	8 (4.4%)
Missing Race Data	7 (8.6%)	3 (1.7%)

* p=0.007 *vs.* Androgen Receiving

**Table 2:** BMI categories of study participants.

Category	Androgen Receiving (n=76)	No Androgen (n=161)
Underweight (BMI<18.5 kg/m^2^)	0 (0.0%)	3 (1.9%)
Normal (BMI 18.5–25 kg/m^2^)	9 (11.8%)	30 (18.6%)
Overweight (BMI 25–30 kg/m^2^)	17 (22.3%)	40 (24.8%)
Obese (BMI>30 kg/m^2^)	50 (65.8%)	88 (54.7%)

p>0.05

**Table 3:** Metabolic profile of study participants.

Variable (Mean ± SD)	Androgen Receiving (n=81)	No Androgen (n=181)
A1c (%)(Ref: 4.4 to 5.6)	7.1 ± 2.3 (n=17)	6.9 ± 2.1 (n=56)
Total Chol (mg/dl) (Ref: <200)	173 ± 38 (n=17)	176 ± 49 (n=52)
HDL Chol (mg/dl) (Ref: >40)	40 ± 8 (n=23)	40 ± 13 (n=62)
LDL Chol (mg/dl) (Ref: <100)	104 ± 32 (n=21)	96 ± 31 (n=58)
Triglycerides (mg/dl) (Ref: <150)	200 ± 189 (n=24)	225 ± 248 (n=63)
SBP (mmHg) (Ref: <140)	144 ± 21 (n=77)	145 ± 25 (n=171)
DBP (mmHg) (Ref: <90)	85 ± 16 (n=77)	89 ± 16 (n=170)
TSH (mcU/ml) (Ref: 0.358 to 3.740)	1.9 ± 1.1 (n=23)	2.1 ± 1.6 (n=63)
Free T4 (ng/dl) (Ref: 0.7 to 1.6)	0.96 ± 0.20 (n=13)	0.97 ± 0.25 (n=24)

p>0.05 for all comparisons

**Table 4:** Glucose homeostasis categories of study participants according to A1C criteria (n=73).

Category	Androgen Receiving (n=17)	No Androgen (n=56)
Normal	5 (6.2%)	21 (11.6%)
Pre-diabetes	4 (4.9%)	12 (6.6%)
Diabetes	8 (9.9%)	23 (12.7%)

p>0.05

## Abbreviations

BMI: Body Mass Index
DBP: Diastolic Blood Pressure
DXA: Dual Emission X-Ray Absorptiometry
KS: Klinefelter Syndrome
SBP: Systolic Blood Pressure
SHBG: Sex Hormone Binding Globulin
T2D: Type 2 Diabetes Mellitus
UNM HSC: University of New Mexico Health Sciences Center
